# The inherent susceptibility of dorsal motor nucleus cholinergic neurons to the neurodegenerative process in Parkinson's Disease

**DOI:** 10.3389/fneur.2012.00189

**Published:** 2013-01-17

**Authors:** A. McCarthy, J. McKinley, T. Lynch

**Affiliations:** Department of Neurology, Dublin Neurological Institute at the Mater Misericordiae University HospitalDublin, Ireland

**A commentary on**

**Calcium entry induces mitochondrial oxidant stress in vagal neurons at risk in Parkinson's disease**

by Goldberg, J. A., Guzman, J. N., Estep, C. M., Ilijic, E., Kondapalli, J., Sanchez-Padilla, J., et al. (2012). Nat. Neurosci. 15, 1414–1421.

What is it about the dopaminergic neurones of the substantia nigra pars compacta (SNpc) and the cholinergic neurones of the dorsal motor nucleus of the vagus (DMV) that makes them particularly prone to the neurodegenerative changes found in Parkinson's disease? If mitochondrial oxidant stress is accepted as an important step in the pathogenesis or Parkinson's disease, are these cell groups inherently prone to this? If they are, is this a potential target for future therapy?

Recent work with the neurons of the SNpc has demonstrated that they are slow autonomous pacemakers with broad spikes (Grace and Bunney, 1984) and that this self-generated activity creates a metabolic stress that may prime these cells for to the deleterious effects of mitochondrial toxins and genetic alterations seen in familial forms of Parkinson's. This form of pacemaking promotes sustained calcium entry through L-type voltage-dependent calcium channels (Nedergaard et al., 1993; Puopolo et al., 2007; Guzman et al., 2010) that is, only weakly buffered by calcium-binding proteins (CaBPs) (Foehring et al., 2009). Goldberg et al. (2012) set out to identify if other neuron subsets might also be vulnerable in this way. They focused their attention on the neurons of the DMV, as it is known that these neurons are amongst the first to show Lewy Body Neurites (LBN) in Parkinson's disease. This marries well with the known early involvement of gut motility in many Parkinson's disease patients and recent experimental work that has demonstrated that alpha-synuclein accumulation in the DMV can be induced by slow gastric lavage of rotenone in rodents (Pan-Montojo et al., 2010). Perforated patch recordings of DMV neurons, taken from the medulla oblongata of choline acetyltransferase-enhanced green fluorescent protein (ChAT-eGFP) BAC transgenic mice, demonstrated autonomous pacemaking activity with broad spikes. This activity continued in the presence of cholinergic, glutamatergic, GABAergic, serotonergic, and adrenergic receptor antagonists. Following on from this, the authors demonstrated that this autonomous pacemaking elevates intracellular calcium concentration. They demonstrated rapid intracellular rise in calcium concentration following a spike and exponential fall in concentration during the interspike interval. A linear relationship between spike duration and calcium concentration was seen. Abolishing spike formation, with the addition of the sodium channel blocker tetrodotoxin (TTX), removed this intracellular calcium fluctuation and provided further evidence against a subthreshold, calcium channel-dependent oscillation. DMV neurons were then demonstrated to have a low intrinsic buffering capacity, meaning their ability to provide an energy neutral means of buffering intracellular calcium is poor. DMV neurons are not known to express detectable levels of calbindin or parvalbumin, two common CaBPs. The authors determined the intrinsic buffering capacity of DMV neurons using the added buffer method (Maravall et al., 2000), to allow for the possibility that these neurons may express other less well-characterized CaBPs. The matrix of DMV mitochondria was shown to be relatively oxidized. When isradipine (L-type calcium channel antagonist) was added, this oxidant stress was diminished, without altering the cells inherent spiking or pacemaking activity. Antagonizing Nav1 sodium channels with TTX (eliminates spiking and calcium entry) and lowering cell ambient temperature (reduces firing rate), both resulted in significantly lower mitochondrial oxidant stress. Increasing spike duration with tetraethylammonium [TEA, an antagonist of the large conductance voltage- and calcium-activated (BK) potassium channel] resulted in a significant increase in mitochondrial oxidant stress. Therefore, spike duration and rate directly influence calcium entry and subsequently the level of mitochondrial oxidant stress. Mutations in DJ-1 are linked with early onset autosomal recessive Parkinson's disease. DJ-1 helps to organize mitochondrial oxidant defences. The authors employed DJ-1^−/−^ mice to explore the effect on mitochondrial oxidant stress. While basal pacemaking, spike width and rate were normal in these mice, basal mitochondrial oxidant stress levels were elevated. This basal mitochondrial stress was improved with pre-incubation with isradipine.

In this important study the authors have highlighted the physiologic similarities between DMV cholinergic neurons and SNpc dopaminergic neurons, proposing a common neuronal phenotype underlying neurodegenerative risk in Parkinson's disease. The inherent pacemaking activity of these cells, via enhanced calcium entry, provides a first “hit” to their mitochondria, which may then be exacerbated further by exposure to environmental toxins or the individual's genetic makeup in rare familial cases of disease. However, DMV cholinergic neurons and SNc dopaminergic neurons appear to differ in their ability to withstand the pathological changes they acquire in Parkinson's disease. Cholinergic neurons appear to meet with this challenge better and animal models suggest they are more resistant to neuronal loss despite accumulation of alpha synuclein pathology (Nedergaard et al., 1993). Therefore, the physiologic similarities described here, do not on their own fully explain neuronal cell death in Parkinson's disease. The study provides further insight as to why individuals using L-type calcium channel blockers for hypertension may be protected from developing Parkinson's disease (Ritz et al., 2010) and points to a potential target for future preventative therapy.
